# Assessment of Risk Factor Progression Due to the COVID-19 Pandemic Among Diagnosed Cases of Hypertension and Diabetes Under the Field Practice Area of Urban Primary Health Centre, Rishikesh, Uttarakhand

**DOI:** 10.7759/cureus.42048

**Published:** 2023-07-17

**Authors:** Santosh Kumar, Naveen Diwan, Ashutosh Mishra, Ajeet S Bhadoria

**Affiliations:** 1 Community and Family Medicine, All India Institute of Medical Sciences Rishikesh, Rishikesh, IND; 2 Epidemiology and Public Health, All India Institute of Medical Sciences Rishikesh, Rishikesh, IND

**Keywords:** smoking tobacco, life style habits, unhealthy diet, diabetes type 2, hypertension and covid-19

## Abstract

Introduction: Hypertension and diabetes were the two pertinent comorbidities of non-communicable disease which was most deadly affected during the COVID-19 pandemic, globally. Therefore, the present study was carried out to assess the progression of risk factors in patients with hypertension and diabetes, and behavioural risk factors during the COVID-19 pandemic.

Material and Methods: A community-based longitudinal study was carried out for a period of one year in patients with pre-existing hypertension and diabetes in the field practise area of urban primary health centres (UPHC) in Rishikesh. The sample size is estimated to be 274, Only 100 study participants could be enrolled due to the COVID-19 pandemic. A convenience sampling method was used. Data were analysed using SPSS Version 23 (IBM Corp., Armonk, NY). Mean ± SD was calculated for continuous variables. The Chi-square test and the Fischer exact test were employed as appropriate to examine the association. To compare the means, the paired “T-test” was utilised.

Result: Mean age of 100 study participants was 56 years ± 11SD. A significant difference (p=0.03) in random blood sugar and diastolic blood pressure was observed before and during the pandemic Proportion of tobacco users and alcoholics was 33% and 22%, respectively. The progression of hypertension and diabetes was reported to be significant among the participants with unhealthy diets and unhealthy lifestyles.

Conclusion: COVID-19 pandemic poses an increase in risk factors like the adoption of unhealthy and sedentary lifestyles, tobacco, and alcohol consumption. All these factors were significantly (p=0.02) associated with the progression of diabetes and hypertension.

## Introduction

In December 2019, Wuhan, China, reported the discovery of coronavirus also known as the severe acute respiratory syndrome coronavirus 2 (SARS-CoV-2). Since then, the virus has spread throughout the world, affecting more than 180 nations [1].

SARS-CoV-2 has spread throughout communities and infected people of various ages, ethnic origins, and genders. An ordinary cold to more serious illnesses including bronchitis, pneumonia, severe acute respiratory distress syndrome, multi-organ failure, and even death are among the clinical symptoms. It is thought that COVID-19 progresses more quickly and severely in those with underlying comorbidities, frequently ending in death. According to reports, comorbidities, male sex, and age more than 60 were reported to be danger risks for death in COVID-19 patients [2]. Hypertension increases the likelihood of developing severe COVID-19, and this finding may be explained by the greater incidence of concomitant conditions in elderly people [2]. According to recent statistics, the most common comorbidities among COVID-19 patients were hypertension, diabetes mellitus, cardiovascular illnesses, and chronic obstructive pulmonary disease. Furthermore, findings show that cardiovascular, hypertensive, and diabetic illnesses are ‘significant’ risk for progression and unsatisfactory outcomes in COVID-19 individuals [2]. COVID-19 lockdown might have increased the risk of cardiovascular disorder (CVDs) by reduced physical activity and an increase in unhealthy behaviour such as smoking, drinking alcohol and unhealthy food eating. Second-hand smoke also increases CVD risk factors by 30% [3]. Due to the COVID-19 pandemic and subsequent lockdown, people with comorbidities like hypertension and diabetes were unable to access health facilities, and they developed an increase in high-risk behaviours. This study was conducted with the objective to assess the progression of risk factors of hypertension and diabetes during the COVID-19 pandemic.

It has been witnessed in various studies that interruption of services for non-communicable diseases (NCDs) during the COVID-19 pandemic, worldwide was a potential public health problem. Disruption of services affected largely those who were already living with the NCDs. This study was also conducted to create a reflection of public health system enforcement for other disease of priority during any emergency. Health system preparedness and building bounce back strategy for NCDs, during and after the crisis, government and policymakers need to commit and ensure that people living with NCDs should not feel the disruption in essential and primary healthcare services.

## Materials and methods

This was a longitudinal community-based study conducted in the field practice area of Urban Primary Health Centre (UPHC) Rishikesh for a period of one year. All the participants from the age group of 30-70 years, who have previously documented records before the first wave of the COVID-19 pandemic (March 2020), a prescription from a registered medical practitioner for diabetes or hypertension or regular or irregular medication for both hypertension and diabetes, irrespective of their medication consumption status were included in the study. Patients with new onset symptoms and no previous records of hypertension and diabetes, severely ill patients, and who required emergency care were excluded from this study.

According to Voigt et al. [4], progression for diabetes was found to be 26% with a relative precision of 20%, and the sample size was calculated at 274, by using the formula, n= z2P (1-P)/d2. Due to the COVID-19 pandemic desired sample size could not be achieved. Only 100 participants were recruited after permission from the institutional ethical committee. A convenience sampling technique was used. This study was conducted during the second wave of the COVID-19 pandemic. The study was planned in mid-September 2020 when the first wave of COVID-19 started declining. At that moment, the sample size calculated was 274 without considering the assumption of the resurgence of the COVID-19 second wave. Data collection started from January to April 2021, which was the period of resurgence of COVID-19 second wave. This was a community-based study, keeping in mind the feasibility issue to conduct this study due to this second wave, Institution's research review board has been requested to reduce the sample size to 100 and ethical permission for the same was taken.

The data were entered into MS Excel sheet 2013 for coding and to check for any missing values or duplication of entries. Then it was exported to IBM SPSS version 23.0 (IBM Corp., Armonk, NY) for data analysis. Mean ± SD was calculated for continuous variables. The Chi-square test and the Fischer exact test were employed as appropriate to examine the association. To compare the means, the paired ‘T-test’ was utilised.

## Results

The mean age (in years) of 100 research participants was 56 years ± 11 SD. The proportion of female participants was 58%. The majority (72%) of the study participants belonged to the nuclear family. As per the modified Kuppuswamy socioeconomic classification 57% participants belong to the upper lower class (Table 1) [5].

**Table 1:** Distribution of socio-demographic characteristics among study participants (N=100) *According to Modified Kuppuswamy Scale (Updated Kuppuswamy scale for year 2020 were obtained using ACPI)

Socio-demographic Variable	Sub-categories	Frequency (%)
Age groups (years)	30-39	4 (4)
	40-49	22 (22)
	50-59	27 (27)
	60-70	47 (47)
Gender	Male	42 (42)
	Female	58 (58)
Family Type	Joint	28 (28)
	Nuclear	72 (72)
Kuppuswamy Socio-economic status	Upper middleclass	10 (10)
	Lower middle class	33 (33)
	Upper lower	57 (57)
Disease status	Hypertension	27 (27)
	Diabetes	48 (48)
	Both hypertension and diabetes	25 (25)

A significant difference (p<0.05) in random blood sugar (RBS) and diastolic blood pressure (BP) was observed among the participants before March 2020, initiation of the first wave and during the pandemic till the data collection was going on from January to April 2021. Rise in BP and blood sugar among the majority of study participants during the pandemic indicate either inaccessibility towards primary healthcare services or inadequate self-care due to the threat of pandemic. A total of one year spent with a sedentary lifestyle due to fear of moving out, physical inactivity and stress may be cause of this progression of diabetes. Weight and BMI were also affected but changes were not significant (Table 2).

**Table 2:** Comparison of random blood sugar and blood pressure of participants before and during the pandemic

Variables	Mean ± Standard deviation	P-value (Paired T-test)
Random blood sugar (mg/dL)		
Before pandemic	184.1 ± 25.3	0.003
During pandemic	213.8 ± 81.6	
Systolic blood pressure (mmHg)		
Before pandemic	139.5 ± 14.2	0.51
During pandemic	144.7 ± 23.4	
Diastolic blood pressure (mmHg)		
Before pandemic	86.81 ± 7.5	0.045
During pandemic	89.15 ± 8.5	

The proportion of tobacco users and alcoholic were 33% and 22%, respectively. Majority (75%) of tobacco and alcohol consumption (81%) was increased during the COVID-19 pandemic. Increased consumption may be attributed to loneliness and poor social alliances due to the lockdown during the pandemic (Table 3).

**Table 3:** Distribution of substance use among participants during COVID-19 pandemic (N=100)

Variables		N (%)	Increased consumption	Decreased/remain same consumption
Tobacco users	Yes	33 (33)	25 (75.8%)	8 (24.2%)
	No	67 (67)	0	0
Alcoholics	Yes	22 (22)	18 (81.8%)	4 (18.2%)
	No	78 (78)	0	0

Progression of hypertension and diabetes was reported to be significant among the participants with unhealthy diet and unhealthy lifestyle (Table 4). Adoption of unhealthy lifestyle during pandemic was one of the pertinent causes to increased number of CVDs. Common unhealthy practices during pandemic were physical inactivity, irregular diet, substance use, etc.

**Table 4:** Association of factors responsible for progression of hypertension and diabetes during the pandemic (N=100) For values <5 Fischer exact value is use.

Variables	Progression		No progression	P-value (Chi-square)
Access to Medical care				
No Access	1 (100%)		0 (0%)	1.0
Access	63 (63.64%)		36 (36.36%)	
Unhealthy diet				
Yes	28 (84.85%)		5 (15.15%)	0.002
No	36 (53.73%)		31 (46.27%)	
Unhealthy lifestyle habits				
Yes	15 (100%)		0 (0%)	0.001
No	49 (57.65%)		36 (42.35%)	
Financial crisis				
Yes	12 (60%)	8 (40%)		0.66
No	52 (65%)	28 (35%)		

Progression in diabetes and hypertension was contributed by both due to common unhealthy practices during pandemic and continuum of care was also interrupted at primary healthcare. Usually, hypertension and diabetic patients having their regular visit to their physician for monitoring, this visit to primary care physician or consultant became difficult and interrupted due to patient fear from COVID-19 transmission exclusively at hospital areas during and after the first wave of COVID-19 pandemic from March 2020 to September 2020. Urban slums population were not well exposed to telemedicine services due to technical issues. However, it was started very late in this area and was not well accepted by this population.

## Discussion

The unprecedented event of COVID-19 pandemic proved to be a potential challenge for public health which leads to an exponential increase in morbidity and mortality due to the SARS-CoV-2 virus. The morbidity due to the SARS-CoV-2 virus is attributed to the pre-existing NCD among the exposed persons. This study was done during the beginning year of the COVID-19 pandemic with the aim to determine the factors associated with the progression of risk factors of NCD during the COVID-19 pandemic.

Total 100 people with a mean age of 56 years ± 11 SD participated in this study. In the present study, the proportion of female participants was 58% and the majority (72%) of them belong to the nuclear family. As per the modified Kuppuswamy socioeconomic classification, 57% of the study participants belong to the upper-lower class. Among the participants, the proportion of diabetes, hypertension and both diabetes with hypertension was 48%, 25% and 23%, respectively.

A significant difference in RBS and BP was observed among the participants before and during the pandemic (p<0.05). Both RBS and BP were found to be raised during a pandemic. These findings might be because of the unavailability of routine consultation of patients with the treating doctors, non-compliance to regular medicine intake, psychosocial issues, anxiety, and unhealthy lifestyles due to the COVID-19 pandemic. Similar findings were observed in studies by Patel et al., Laffin et al., Shah et al. and Khare et al. [6-9].

The proportion of tobacco users and alcoholics were 33% and 22%, respectively. During the COVID-19 pandemic, out of total tobacco users, 75.8% increased tobacco consumption and the majority (80%) of alcoholics increased consumption. This might be because of sudden lockdown, increased stress, loss of jobs and more leisure time during the pandemic. A similar study conducted in 2022 reported that 52% were currently using alcohol, while 63% were using tobacco and about 28% of participants reported increasing the frequency and amount of substance intake during the COVID-19 lockdown period [10]. Various studies observed similar results [10-13]. The risk of cardiovascular diseases has been reported to be raised during the pandemic due to increasing substance use and the adoption of a sedentary lifestyle.

The progression of hypertension and diabetes was observed to be significantly linked among the participants with unhealthy diets and unhealthy lifestyles. In contrast, Azzouzi et al. found that the presence of NCD was associated with a 24% greater likelihood of positive changes in daily fruit and vegetable consumption and a 46% greater likelihood of reducing tobacco use. The presence of NCDs was shown to be adversely and substantially linked with 27% greater odds of reduced night-time sleep hours and 27% higher odds of reporting physical health deterioration [14]. Because of the pandemic and lockdown, the observation of the current study enforces the adoption of unhealthy diets and lifestyles with substance use which were the risk factors for the progression of NCDs.

Limitation

Convenience sampling was done to select the study participants and the sample size was also reduced to make the study feasible. Due to the less sample size study results may not be generalised to the larger population. As the progression of Diabetes and hypertension is multifactorial, in this study, only some factors due to the COVID-19 pandemic during the first wave have been mentioned. Many other confounders may be present with the results as other routine factors, such as panic attacks, stress and depression, for the progression of these diseases have not been mentioned.

## Conclusions

The COVID-19 pandemic poses an increase in risk factors like the adoption of unhealthy and sedentary lifestyles, tobacco and alcohol consumption. All these factors were significantly associated with in progression of Diabetes and hypertension. 

Hypertension and diabetes are lifestyle diseases. Lifestyle modifications are the mainstay for the part of management. Our aim was to explore that due to difficult access to hospitals, the workplace, online work culture, and substance use have influenced badly the progression of diabetes and hypertension. The fear of going outside even in the morning walk was a concern of people, due to this physical inactivity they land up with obesity. An unhealthy lifestyle and fear to visit a physician in the hospital during the first and second wave of the COVID-19 pandemic was the pertinent reason for the progression of this disease.

However, the gatekeeping system of primary care has been evidenced to be disrupted during the COVID-19 pandemic which was a potential threat for regular monitoring and prevention of lifestyle diseases. Progression in risk factors for NCDs has also contributed to mortality due to the COVID-19 pandemic. Poor accessibility to the health care system, Panic and fear of transmission in hospitals affected health seeking behaviour of patients with chronic disease. Intensive awareness programmes and community mobilisation for a healthy lifestyle and hassle-free health-seeking behaviour for these morbidities need to be advocated during post COVID-19 period.
